# Comprehensive analysis of the amino acid metabolism-related gene signature for prognosis, tumor immune microenvironment, and candidate drugs in hepatocellular carcinoma

**DOI:** 10.3389/fimmu.2022.1066773

**Published:** 2022-12-13

**Authors:** Yue Li, Huanye Mo, Siying Jia, Jun Wang, Ying Ma, Xin Liu, Kangsheng Tu

**Affiliations:** ^1^ Department of Hepatobiliary Surgery, The First Affiliated Hospital of Xi’an Jiaotong University, Xi’an, China; ^2^ Department of Emergency and Critical Care Medicine, The First Affiliated Hospital of Xi’an Jiaotong University, Xi’an, China; ^3^ Department of Cardiovascular Medicine, Xi’an No.3 Hospital, The Affiliated Hospital of Northwest University, Xi’an, China; ^4^ The Key Laboratory of Tumor Molecular Diagnosis and Individualized Medicine of Zhejiang Province, Zhejiang Provincial People’s Hospital, Affiliated People’s Hospital, Hangzhou Medical College, Hangzhou, China

**Keywords:** hepatocellular carcinoma, amino acid metabolism, prognosis, immune microenvironment, drug sensitivity

## Abstract

**Introduction:**

Metabolic rewiring satisfies increased nutritional demands and modulates many oncogenic processes in tumors. Amino acid metabolism is abnormal in many malignancies. Metabolic reprogramming of amino acids not only plays a crucial role in sustaining tumor cell proliferation but also influences the tumor immune microenvironment. Herein, the aim of our study was to elucidate the metabolic signature of amino acids in hepatocellular carcinoma (HCC).

**Methods:**

Transcriptome profiles of HCC were obtained from the TCGA and ICGC databases. Based on the expression of amino acid metabolism-related genes (AAMRGs), we clustered the HCC samples into two molecular subtypes using the non-negative matrix factorization algorithm. Then, we constructed the amino acid metabolism-related gene signature (AAMRGS) by Cox regression and LASSO regression. Afterward, the clinical significance of the AAMRGS was evaluated. Additionally, we comprehensively analyzed the differences in mutational profiles, immune cell infiltration, immune checkpoint expression, and drug sensitivity between different risk subgroups. Furthermore, we examined three key gene expressions in liver cancer cells by quantitative real-time PCR and conducted the CCK8 assay to evaluate the influence of two chemotherapy drugs on different liver cancer cells.

**Results:**

A total of 81 differentially expressed AAMRGs were screened between the two molecular subtypes, and these AAMRGs were involved in regulating amino acid metabolism. The AAMRGS containing GLS, IYD, and NQO1 had a high value for prognosis prediction in HCC patients. Besides this, the two AAMRGS subgroups had different genetic mutation probabilities. More importantly, the immunosuppressive cells were more enriched in the AAMRGS-high group. The expression level of inhibitory immune checkpoints was also higher in patients with high AAMRGS scores. Additionally, the two AAMRGS subgroups showed different susceptibility to chemotherapeutic and targeted drugs. In vitro experiments showed that gemcitabine significantly reduced the proliferative capacity of SNU449 cells, and rapamycin remarkedly inhibited Huh7 proliferation. The five HCC cells displayed different mRNA expression levels of GLS, IYD, and NQO1.

**Conclusions:**

Our study explored the features of amino acid metabolism in HCC and identified the novel AAMRGS to predict the prognosis, immune microenvironment, and drug sensitivity of HCC patients. These findings might help to guide personalized treatment and improve the clinical outcomes of HCC.

## Introduction

According to the global cancer statistics of 2020, hepatocellular carcinoma (HCC) is still one of the top 10 most common malignancies and the third leading cause of cancer-related death (1). The high mortality rate of HCC is mainly due to the fact that symptoms are often insidious until the late stage, and the diagnosis is delayed (2). In such cases, the majority of patients with advanced HCC are not amenable to curable surgical treatment. Over the past decade, multi-targeted tyrosine kinase inhibitors, including sorafenib and lenvatinib, have been regarded as the first-line treatment drug for late-stage HCC patients (3). In recent years, immune checkpoint inhibitors have been used as second-line drugs with the development of immunotherapy (4). However, the survival time of HCC patients is only extended by a few months, and the overall prognosis is still unsatisfactory. Exploring the intrinsic features of HCC and identifying a new predictive biomarker are imperative to improving the clinical efficacy and prognosis of HCC patients.

Metabolic reprogramming is a well-known hallmark of cancer. When suffering from a severe nutritional crisis, the metabolism of tumor cells is significantly altered to meet their growth requirements (5). In recent decades, increasing studies have revealed the alteration of metabolic profiling in cancers (6–8). The activation of abnormal metabolic pathways not only causes persistent proliferation of tumor cells but also affects the tumor microenvironment (9). Amino acid metabolism is critical for maintaining redox homeostasis, biosynthesis, providing energy-producing metabolic intermediates, and modulating epigenetic modifications (10). Recently, many studies have emphasized the vital role of amino acid metabolism reprogramming in tumors. For instance, reprogramming glutamine metabolism provides a carbon source for *de novo* synthesis of fatty acids to support tumor growth, leading to liver cancer cells being resistant to sorafenib (11). Tong et al. reported a close relationship between impaired tyrosine catabolism and poor prognosis in HCC (12). Silencing tyrosine catabolic enzyme in liver cells increases cellular dependence on glutamine. Additionally, amino acid metabolism has essential effects on the immune response. As the predominant immune effector cells, the functional status of T cells is related to anti-tumor immunity. Some studies found that elevated 5-methylthioadenosine and S-adenosylmethionine drive T cells from effector to depleted state (13). Targeting the methionine recycling pathway may be a feasible therapeutic strategy to enhance immunity in HCC. Besides, enhanced glutamine metabolism driven by HMGB1 promotes tumorigenesis and hampers immunotherapy efficacy (14). Thus, a better understanding of the amino acid metabolic profile in HCC is necessary to improve prognosis and treatment sensitivity for patients.

Here, we focused on the characteristics of amino acid metabolism in HCC and systematically analyzed its clinical significance for immunotherapy and molecular therapeutics. First, we identified two molecular subtypes based on the expression of amino acid metabolism-related genes (AAMRGs) and compared the different molecular features between the subtypes. Then, the amino acid metabolism-related gene signature (AAMRGS) was constructed for prognosis prediction. Further, we explored the implications of the AAMRGS on mutational profile, immune microenvironment, and drug sensitivity. The results suggested that the novel AAMRGS is of significant value in improving outcomes and therapeutic efficacy of HCC.

## Materials and methods

### Data collection and processing

The genomic data of RNA sequencing and clinical information for HCC were acquired from the TCGA (https://portal.gdc.cancer.gov/) and ICGC databases (https://dcc.icgc.org/). Samples with survival time ≥ 30 days were included in the study. To ensure the validity of the results, we took the TCGA dataset as the training cohort and the ICGC dataset as the validation cohort.

### Clustering analysis

We downloaded the set of AAMRGs (REACTOME_METABOLISM_OF_AMINO_ACIDS_AND_DERIVATIVES) from the Molecular Signatures Database (MSigDB, version 7.5.1). After overlapping with TCGA-LIHC RNA sequencing data, we acquired the expression profile of 374 genes related to amino acid metabolism in all liver cancer samples. Before clustering analysis, AAMRGs were subjected to the univariate Cox regression analysis to obtain the genes associated with the overall survival (OS) (p < 0.01). Then, we performed the non-negative matrix factorization (NMF) consensus clustering with the R package NMF, which was based on the expression of AAMRGs in each sample. The optimal k value for the clustering was determined when the cophenetic correlation coefficient started to fall. Besides, we conducted the principal component analysis (PCA) to assess whether samples were grouped correctly.

### Gene set variation analysis

The gene set variation analysis (GSVA) was used to compare the enrichment of KEGG and hallmark gene sets in clustering groups.

### Differentially expressed AAMRGs

The limma package was applied to screen the differentially expressed AAMRGs between the clustered subgroups. The |log2 fold change (FC) | > 1 and false discovery rate (FDR) < 0.05 were set as filtering criteria. To further understand the potential molecular functions of genes, we conducted the GO and KEGG enrichment analysis.

### Construction and verification of the AAMRGS

Univariate Cox regression analysis was performed to screen out the differentially expressed AAMRGs related to prognosis (p < 0.05) in the training cohort. Next, prognostic AAMRGs were further screened by the least absolute shrinkage and selection operator (LASSO) regression analysis to eliminate over-fitting between genes. Then, we obtained AAMRGs and their corresponding regression coefficients included in the final signature construction using the multivariate Cox regression analysis. Based on this, AAMRGS score = expression_gene1_ × coefficient_gene1_ + expression_gene2_ × coefficient_gene2_ + … + expression_gene(n)_ × coefficient_gene(n)_. We calculated the AAMRGS score of all samples in the training and validation cohorts. According to the median AAMRGS score, patients were stratified into the AAMRGS-low and the AAMRGS-high groups. The Kaplan-Meier (KM) survival analysis was performed to compare the difference in survival time between the two subgroups. To assess the predictive performance of the AAMRGS score, we conducted the time-dependent ROC curve analysis and calculated the area under curve (AUC) value at 1, 3, and 5 years of the AAMRGS. Subsequently, the Cox proportional hazards regression model was used to evaluate the independent prognostic value of the AAMRGS. In addition, we probed the correlation of the AAMRGS score with clinicopathological factors.

### Estimation of mutation status

Somatic mutation data of HCC samples were downloaded from the TCGA portal. We calculated the tumor mutation burden (TMB), which was defined as the total number of somatic gene coding errors, base insertion, deletion, or substitution detected per million bases. The mutation landscape in the AAMRGS-low group and the AAMRGS-high group was created by the R package maftools. Furthermore, we evaluated whether the TMB combined with the AAMRGS was an essential factor influencing survival.

### Immunocyte infiltration and immune function

The CIBERSORT algorithm with 1000 permutations was applied to infer the relative fraction of 22 types of infiltrating immunocytes based on the expression profile of HCC samples (15). Then, we compared the differences in the level of immune cell infiltration between subgroups. According to the 29 immune-related gene sets, the single sample gene set enrichment analysis (ssGSEA) was carried out to evaluate the immune status (16). Subsequently, the analysis results of differences in immune function were visualized by bar graphs.

### Immune checkpoint and immunologic signature

Considering that immune checkpoints are important targets for immunotherapy, we contrasted their expression level in the AAMRGS-low and the AAMRGS-high groups. Besides, we downloaded immunologic signature gene sets from the MSigDB database. Gene set enrichment analysis (GSEA) was performed to acquire the enrichment score of immune features among the two groups.

### Screening for potential drugs

The Cancer Genome Project (CGP) database contains whole-genome gene expression data before drug treatment and the sensitivity of 138 drugs in over 700 cell lines (17). Based on the data from the CGP, we predicted the clinical chemotherapeutic response in different AAMRGS groups using the R package pRRophetic (18), which provides a statistical model to derive the IC50 value of drugs.

### Cell culture

The human normal hepatocyte MIHA and HCC cell lines (Hep3B, Huh7, HepG2, HCCLM3) were derived from our laboratory depository (19). The SNU449 cell line was kindly gifted by Professor Yilei Zhang (Xi’an Jiaotong University). All cells were cultured in the Dulbecco’s Modified Eagle’s Medium (DMEM, CellMax) supplemented with 10% fetal bovine serum (ExCell Bio) and antibiotics (100 μg/mL streptomycin and 100 U/mL penicillin, Gibco) at 37°C with 5% CO2.

### RNA extraction and quantitative real-time PCR

Total RNA was extracted from cells by the Trizol method (Invitrogen). According to the manufacturer’s instruction, 1μg of RNA was reverse transcribed to cDNA using the ABScript III RT Master Mix for qPCR (ABclonal). Then, gene expression levels were measured by Bio-Rad CFX96 real-time system using 2X Universal SYBR Green Fast qPCR Mix (ABclonal). For normalization, GAPDH was used as the internal reference gene. The relative gene expression was calculated using the 2^−ΔΔCt^ method. Primer sequences were listed in **Supplementary Table S1**.

### Drug treatment and cell proliferation assay

HCC cells (5000 cells/well) were seeded in 96-well plates and grown overnight. Then, the cells were treated with rapamycin (10 nM, MCE) or gemcitabine (16 μM, MCE) for 48h. Control cells were treated with DMSO. CCK8 assay (Elabscience) was performed to detect cell proliferation according to the instruction.

### Statistical analysis

In this study, all statistical analyses were completed in R software (version 4.1.1). The Wilcoxon rank-sum test was used to compare the two groups. Multiple group comparison was performed by the Kruskal-Wallis test. The log-rank test was applied to survival analysis. The prognostic value of the AAMRGS was assessed by the Cox regression model. Correlations were performed using Spearman’s rank correlation test. A p-value less than 0.05 was considered significant.

## Results

### Identification of two amino acid metabolism-related molecular subtypes

**Figure 1:**
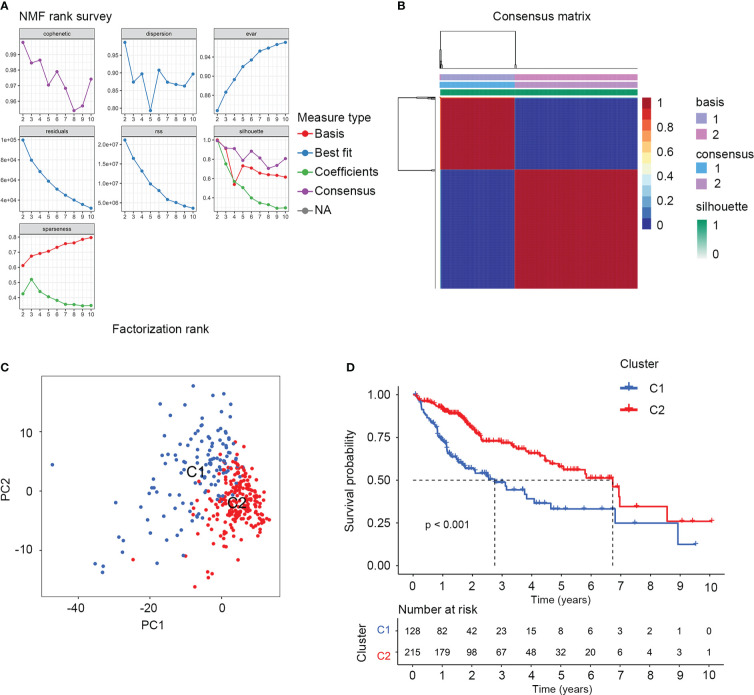
The NMF consensus clustering analysis identified two amino acid metabolism-related molecular subtypes for HCC samples. **(A)** Factorization rank for k = 2 to k = 10. **(B)** The heatmap of consensus clustering matrix when the k=2. **(C)** PCA validated the sample clustering analysis results. **(D)** KM survival analysis of the two molecular subgroups.

There were 374 AAMRGs obtained from the “REACTOME_METABOLISM_OF_AMINO_ACIDS_AND_DERIVATIVES” gene set. In the TCGA cohort, we screened 75 AAMRGs associated with the prognosis through the univariate Cox regression analysis (**Supplementary Table S2**). Then, the HCC samples from the training cohort were subjected to NMF consensus clustering analysis based on the expression matrix of prognostic AAMRGs. As shown in **Figure 1A**, when the value of k was 2, the cophenetic correlation coefficient began to decline. In addition, the heatmap revealed the consensus matrix for k = 2 (**Figure 1B**). Consequently, the HCC samples were separated into two clusters, including C1 (n = 128) and C2 (n = 215). To validate the grouping result of the cluster analysis, we performed the PCA. The samples of two molecular subgroups were clearly separated as in **Figure 1C**. Meanwhile, the KM survival curve showed the difference in OS between the C1 and C2 groups (**Figure 1D**).

### Characteristics of the two molecular subtypes

**Figure 2:**
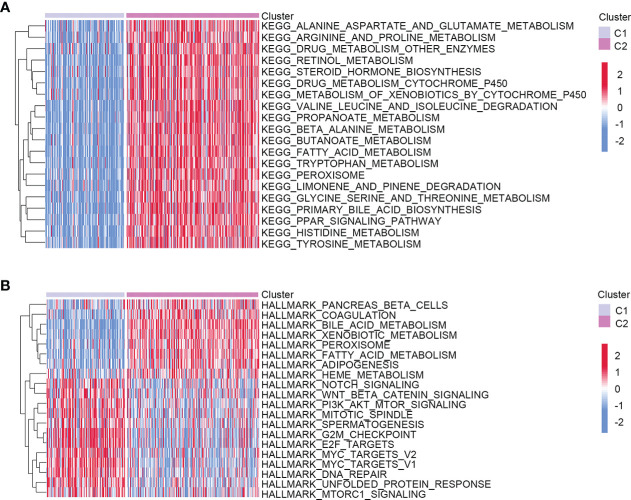
Heatmap showed the molecular characteristics of two different clustering groups. **(A)** KEGG. **(B)** Hallmark gene sets.

To better understand the molecular features of the two clusters, we performed the GSVA to determine KEGG and hallmark gene sets enriched in different clustering groups. As a result, amino acid metabolism-related signaling pathways were more enriched in the C2 group, such as alanine aspartate, glutamate, arginine, and proline metabolism (**Figure 2A**). The enrichment results of hallmark gene sets showed that the metabolism and synthesis of substances were more active in C2 (**Figure 2B**). Therefore, the two clusters had different molecular characteristics and displayed different metabolic states.

### Functional analysis of differentially expressed AAMRGs

There were 81 differentially expressed AAMRGs between C2 and C1 groups. Among them, 52 AAMRGs were upregulated in the C2 group, while 29 AAMRGs were down-regulated (**Supplementary Figure S1**). The GO enrichment results indicated that differentially expressed AAMRGs were mainly involved in the amino acid metabolic process and binding with bioactive substances (**Table 1**). Results of KEGG showed that these genes participated in the metabolism and biosynthesis of amino acids (**Table 2**).

**Table 1:** GO enrichment results of differentially expressed AAMRGS.

Ontology	ID	Description	Count	Adjust p-value
BP	GO:0006520	cellular amino acid metabolic process	53	3.21E-71
BP	GO:1901605	alpha-amino acid metabolic process	46	1.87E-69
BP	GO:0009063	cellular amino acid catabolic process	38	7.95E-64
BP	GO:1901606	alpha-amino acid catabolic process	34	5.50E-58
BP	GO:0016054	organic acid catabolic process	42	3.86E-55
CC	GO:0005759	mitochondrial matrix	23	9.53E-17
CC	GO:0022626	cytosolic ribosome	13	2.05E-14
CC	GO:0044391	ribosomal subunit	13	2.08E-11
CC	GO:0005840	ribosome	13	4.33E-10
CC	GO:0022625	cytosolic large ribosomal subunit	7	5.68E-08
MF	GO:0019842	vitamin binding	13	1.16E-11
MF	GO:0016645	oxidoreductase activity, acting on the CH-NH group of donors	8	3.62E-11
MF	GO:0003735	structural constituent of ribosome	13	6.36E-11
MF	GO:0030170	pyridoxal phosphate binding	9	7.83E-11
MF	GO:0070279	vitamin B6 binding	9	7.83E-11

**Table 2:** KEGG enrichment results of differentially expressed AAMRGS.

ID	Description	Count	Adjust p-value
hsa00260	Glycine, serine and threonine metabolism	15	1.58E-19
hsa01230	Biosynthesis of amino acids	15	3.49E-15
hsa00250	Alanine, aspartate and glutamate metabolism	12	5.12E-15
hsa00270	Cysteine and methionine metabolism	11	8.69E-12
hsa00220	Arginine biosynthesis	8	1.27E-10
hsa00380	Tryptophan metabolism	9	1.07E-09
hsa00330	Arginine and proline metabolism	9	5.85E-09
hsa03010	Ribosome	12	1.40E-07
hsa00350	Tyrosine metabolism	6	5.18E-06
hsa05171	Coronavirus disease - COVID-19	12	7.21E-06
hsa00340	Histidine metabolism	5	7.21E-06
hsa00310	Lysine degradation	7	7.83E-06
hsa00280	Valine, leucine and isoleucine degradation	6	2.07E-05
hsa00630	Glyoxylate and dicarboxylate metabolism	5	2.89E-05
hsa01240	Biosynthesis of cofactors	9	4.01E-05

### Development of the AAMRGS and evaluation of its prognostic significance

**Figure 3:**
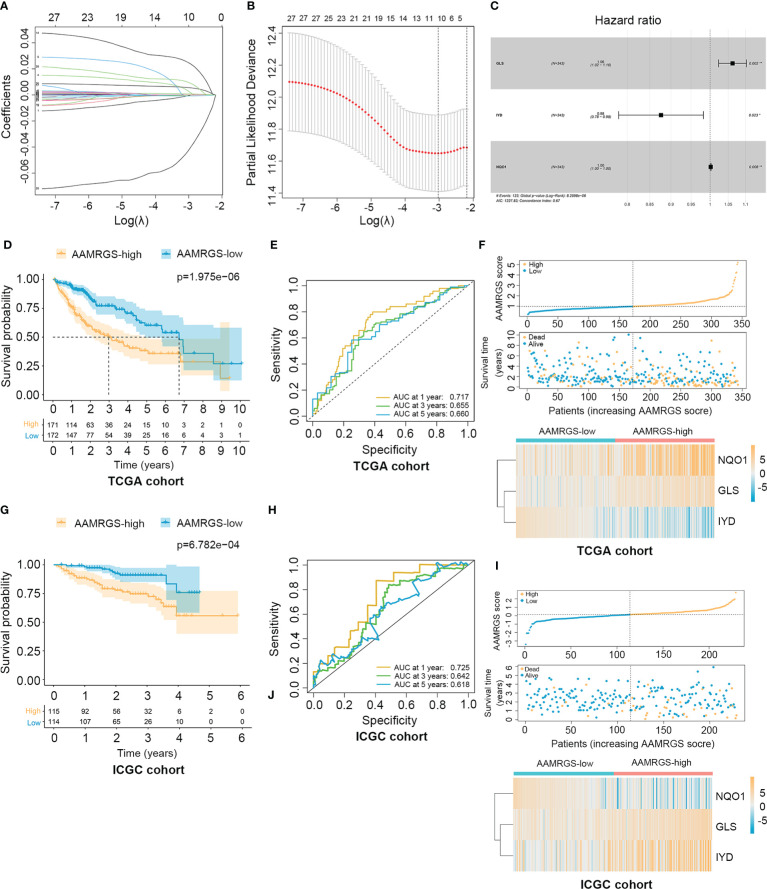
Construction of the AAMRGS and assessment of its prognosis value in TCGA and ICGC cohorts. **(A)** Variation of LASSO coefficients in different tuning parameters (λ). **(B)** Partial likelihood deviance was plotted against log (λ), where λ was the tuning parameter. **(C)** Forest plot of three AAMRGs screened by multivariate Cox regression analysis. **(D)** Difference in survival time between the AAMRGS-high and the AAMRGS-low groups in the TCGA cohort. **(E)** ROC analysis for the AAMRGS at one, three, and five years in the TCGA cohort. **(F)** The distribution of the AAMRGS score, survival status of patients, and expression level of three AAMRGs between the two AAMRGS subgroups in the TCGA cohort. **(G)** KM survival analysis for the ICGC cohort. **(H)** ROC curves for the ICGC cohort. **(I)** The distribution of the AAMRGS score, survival status of patients, and expression level of three AAMRGs between the two AAMRGS subgroups in the ICGC cohort.

**Figure 4:**
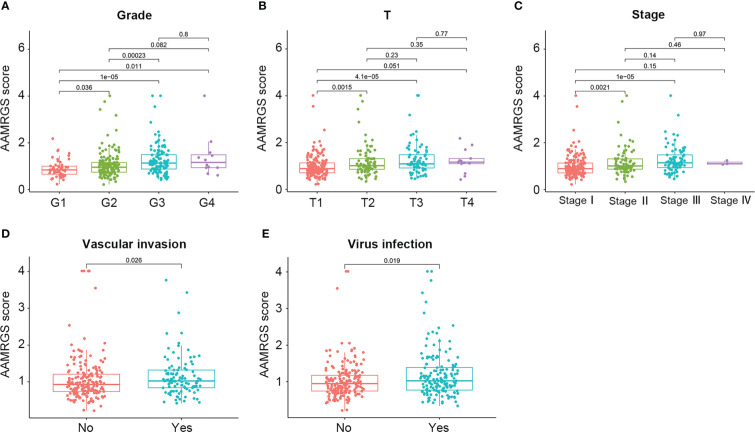
Correlation of the AAMRGS score with clinicopathological features in the TCGA-HCC cohort. **(A)** Pathological grade. **(B)** T staging. **(C)** Clinical staging. **(D)** Vascular invasion. **(E)** Virus infection.

To construct the AAMRGS, first, we screened 28 differentially expressed AAMRGs associated with OS in the training cohort using the univariate Cox regression analysis (**Supplementary Table S3**). Then, by the LASSO algorithm and multivariate Cox regression analysis, three AAMRGs were selected as optimal prognosis-related genes to build the final model (**Figures 3A–C**). After that, the AAMRGS score of each patient in the training and validation cohorts was calculated. Based on the following formula: AAMRGS score = GLS expression × 0.061 + IYD expression × (-0.132) + NQO1 expression × 0.002. The median value of the AAMRGS score was served as the cut-off to classify patients into two subgroups, including the AAMRGS-low and the AAMRGS-high groups. To assess the predictive value of the AAMRGS, we performed the KM and ROC analysis. As shown in **Figure 3D**, the OS and median survival were significantly short in the AAMRGS-high group compared with the AAMRGS-low group. Additionally, we conducted the subgroup survival analysis to exclude the influence of other clinicopathological parameters. The results of subgroup analysis also demonstrated that patients with high AAMRGS scores had poor prognoses (**Supplementary Figure S2**). Besides this, the time-dependent ROC showed that the area under the curve (AUC) value was 0.717, 0.655, and 0.660, respectively, at one, three, and five years (**Figure 3E**). Compared to other clinical factors, the AAMRGS score remained superior for predicting the prognosis (**Supplementary Figure S3**). More importantly, the validation cohort results were consistent with the above results (**Figures 3G, H**). In **Figures 3F, I**, the plots displayed the distribution of the AAMRGS score, survival status, and three important genes in the TCGA and ICGC cohorts. To further assess the independence of the AAMRGS in predicting prognosis, we performed univariate and multivariate Cox regression analyses, which suggested that the AAMRGS score was an independent prognostic factor (**Table 3**). Furthermore, the AAMRGS score was closely correlated with clinicopathological features, including pathological grade, T staging, clinical-stage, vascular invasion, and virus infection (**Figure 4**). That viewed, the AAMRGS was a risk factor and affected the progression of HCC.

**Table 3:** Univariate and multivariate Cox regression analyses of the AAMRGS score in the TCGA.

Variable	Univariate analysis			Multivariate analysis		
	HR	95% CI	P-value	HR	95% CI	P-value
AAMRGS score	4.163	2.249-7.707	5.657E-06	3.224	1.550-6.704	0.002
Age	0.998	0.979-1.016	0.799	1.003	0.984-1.023	0.731
Gender	0.819	0.488-1.376	0.451	1.232	0.672-2.258	0.500
Grade	0.917	0.654-1.286	0.617	1.113	0.756-1.637	0.588
Clinical Stage	2.157	1.649-2.821	1.988E-08	0.738	0.254-2.148	0.578
T	2.098	1.634-2.694	6.461E-09	2.436	0.927-6.400	0.071
N	2.290	0.557-9.419	0.251	1.907	0.317-11.467	0.481
M	4.401	1.371-14.132	0.013	1.233	0.312-4.872	0.765
Fibrosis	0.357	0.201-0.633	0.000	0.580	0.292-1.149	0.118
Vascular invasion	0.950	0.506-1.784	0.873	0.672	0.334-1.352	0.265
Virus infection	3.442	2.039-5.810	3.713E-06	2.031	1.132-3.644	0.018

HR, hazard ratio; 95%CI, 95% confidence interval.

### Mutational profile and biological characteristics of different AAMRGS subgroups

**Figure 5:**
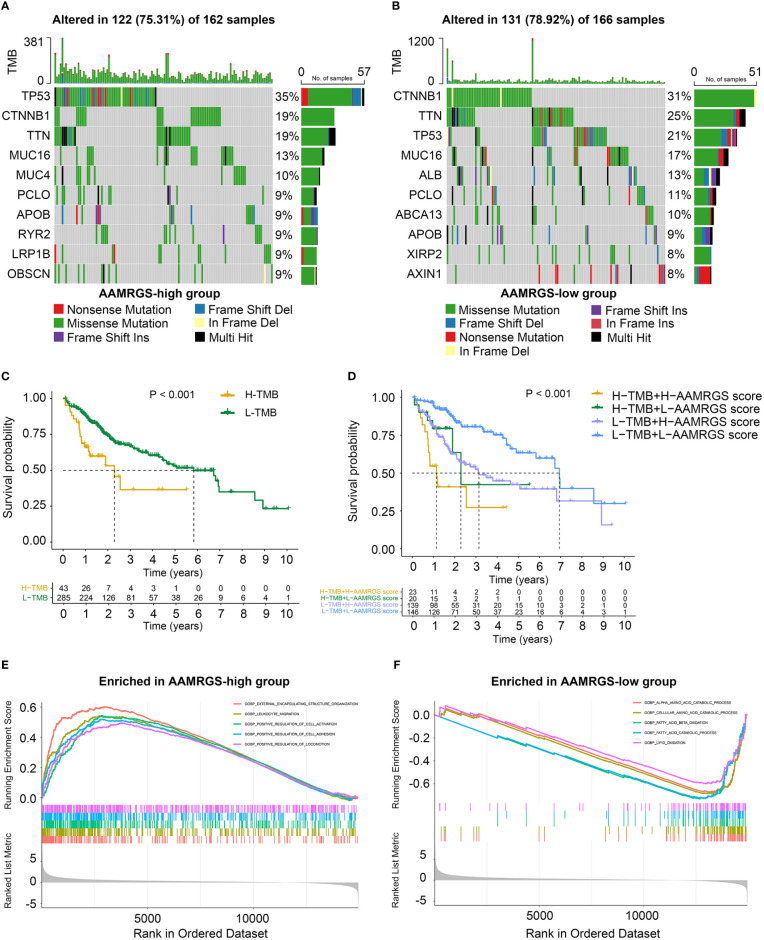
Mutational landscape and biological characteristics in the AAMRGS-high and AAMRGS-low groups. **(A)** Top 10 genes with high mutation probability in the AAMRGS-high group. **(B)** Top 10 genes with high mutation probability in the AAMRGS-low group. **(C)** KM survival analysis of the TMB. **(D)** Influence of the AAMRGS score combined with TMB on OS. **(E)** Enrichment of gene sets associated with biological processes in the AAMRGS-high group. **(F)** Enrichment of gene sets associated with biological processes in the AAMRGS-low group.

Genomic mutations are a pathogenic and defining characteristic of all cancers. The accumulation of genetic mutations can affect the function phenotype and drive tumor development. We found that missense mutation was the most common type of somatic mutation. Besides this, in the AAMRGS-high group, the top ten genes in terms of mutation probability were TP53, CTNNB1, TTN, MUC16, MUC4, PCLO, APOB, RYR2, LRP1B, and OBSCN, while the most frequently mutated genes were CTNNB1, TTN, TP53, MUC16, ALB, PCLO, ABCA13, APOB, XIRP2, and AXIN1 in the AAMRGS-low group (**Figures 5A, B**). Somatic mutations have an influence on clinicopathological outcomes and prognosis. Then, according to the number of gene mutations in each sample, we calculated the TMB and explored its effect on the prognosis. The KM survival analysis showed that HCC patients with high TMB had a shorter survival time than patients with lower TMB (**Figure 5C**). Moreover, the group with high TMB and high AAMRGS scores had the worst prognosis compared with other groups. The patients with low TMB and low AAMRGS scores had the best outcome (**Figure 5D**). These findings indicated that the genetic mutations differed in the two AAMRGS subgroups, and the TMB combined with the AAMRGS score was an important factor affecting survival.

Next, we used the GSEA to gain further biological insight into the molecular processes of the different AAMRGS subgroups. We found that the biological processes of the AAMRGS-high group were mainly enriched in external encapsulating structure organization, leukocyte migration, positive regulation of cell activation, adhesion, and locomotion (**Figure 5E**). In contrast, the gene sets of the AAMRGS-low group were predominantly enriched in the amino acid catabolic process, fatty acid, and lipid oxidation (**Figure 5F**). These results indicated the different biological processes between the two AAMRGS subgroups.

### Immunocyte infiltration and immune function in different AAMRGS subgroups

**Figure 6:**
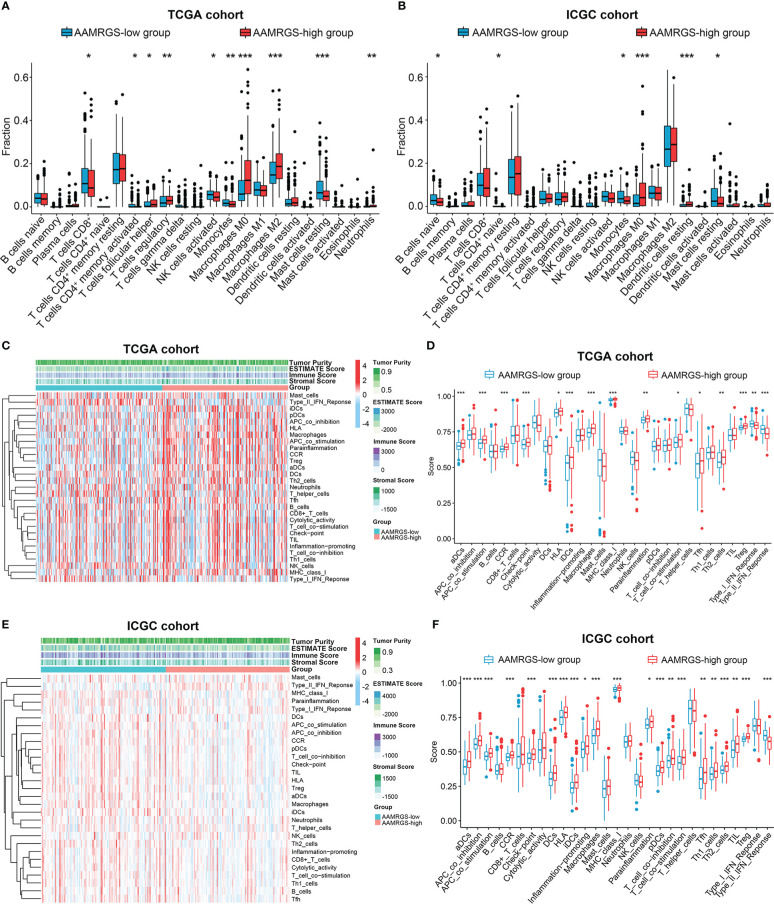
The differences in immune cell infiltration and immune function between the different AAMRGS subgroups. **(A)** The abundance of immune cells in the AAMRGS subgroups in the TCGA cohort. **(B)** The abundance of immune cells in the AAMRGS subgroups in the ICGC cohort. **(C)** Heatmap of the 29 immune-related gene sets enriched among the AAMRGS-high and AAMRGS-low groups in the TCGA cohort. **(D)** Differences in immune function among the two AAMRGS subgroups in the TCGA cohort. **(E)** Heatmap of the 29 immune-related gene sets enriched among the AAMRGS-high and AAMRGS-low groups in the ICGC cohort. **(F)** Differences in immune function among the two AAMRGS subgroups in the ICGC cohort. * P < 0.05, ** P < 0.01, *** P < 0.001.

The functional status of the immune microenvironment is now understood to be inextricably linked to metabolism, which is vital for the survival, proliferation, and activation of immune cells (20). Hence, we explored the relative proportions of 22 immunocytes in different AAMRGS subgroups *via* the CIBERSORT algorithm. Our results showed that the abundances of CD8^+^ T cells, activated memory CD4^+^ T cells, activated natural killer (NK) cells, monocytes, and resting mast cells were higher in the AAMRGS-low group. Furthermore, the infiltration of follicular helper T (Tfh) cells, regulatory T cells (Tregs), M0 macrophages, M2 macrophages, and neutrophils was significantly higher in the AAMRGS-high group (**Figure 6A**). Interestingly, the results of the ICGC cohort indicated that naive B cells, naive CD4^+^ T cells, monocytes, and resting mast cells were more abundant in the AAMRGS-low group, while M0 macrophages and resting dendritic cells (DCs) infiltrated more in the AAMRGS-high group (**Figure 6B**).

Additionally, based on the 29 immune-related gene sets, we characterized the immune landscape of different AAMRGS subgroups (**Figures 6C, E**). As a result, the activity and abundance of the pathway, molecular function, and immune cells displayed significant differences in the two subgroups (**Figures 6D, F**). There were more infiltrating immune cells and immune-modulating molecules in the AAMRGS-high group. Intriguingly, interferon (IFN) response activity was visibly higher in the AAMRGS-low group. These findings suggested that amino acid metabolism influenced the tumor immune microenvironment (TIME) and functional status.

### The differences in immune checkpoints between the two AAMRGS subgroups

**Figure 7:**
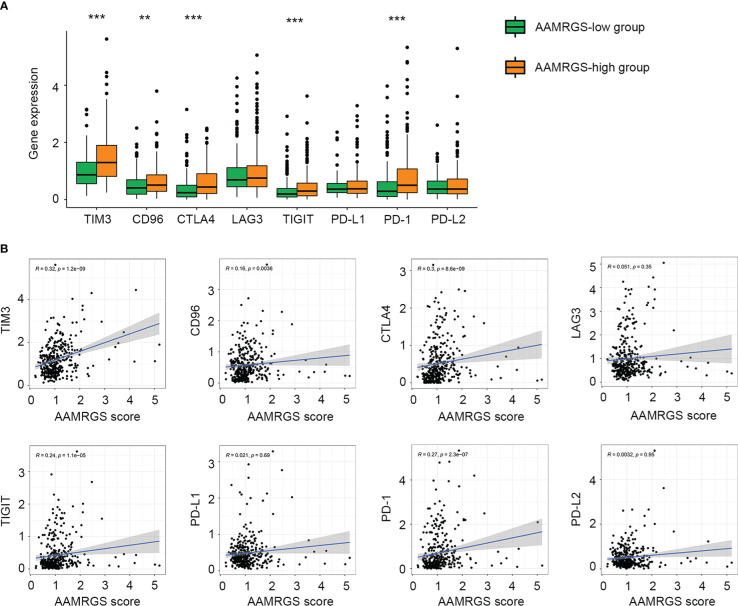
Association of the AAMRGS scores with immune checkpoints. **(A)** Differential expression of immune checkpoints between the AAMRGS-high and AAMRGS-low groups. **(B)** Correlation analysis of immune checkpoints and the AAMRGS scores. **P < 0.01, ***P < 0.001.

Given the importance of immune checkpoints in anti-tumor immune response and immunotherapy, we further compared the expression level of immune checkpoints among the two AAMRGS subgroups. As a result, in the TCGA cohort, TIM3, CD96, CTLA4, TIGIT, and PD1 displayed significantly higher expression in the AAMRGS-high group than in the AAMRGS-low group (**Figure 7A**). Meanwhile, correlation analysis indicated that the expression of these immune checkpoints was positively associated with the AAMRGS score (**Figure 7B**). Similar results were acquired from the ICGC cohort (**Supplementary Figure S4**).

### Drug sensitivity in the different AAMRGS subgroups

**Figure 8:**
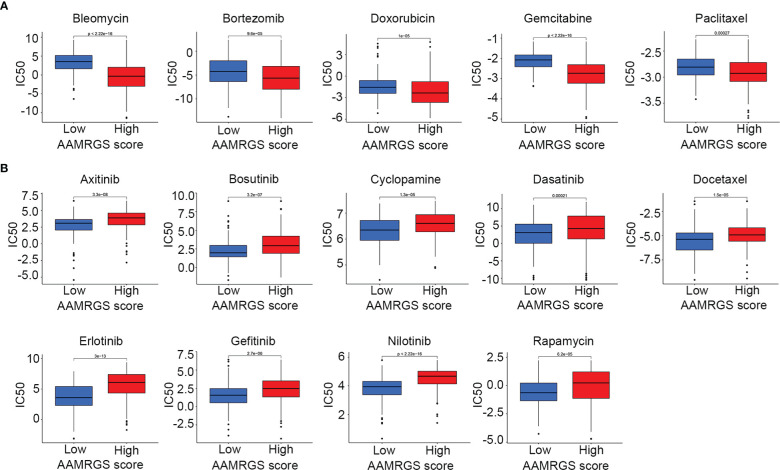
Drug sensitivity in the different AAMRGS subgroups. **(A)** Drugs with high sensitivity in the AAMRGS-high group. **(B)** Drugs with high sensitivity in the AAMRGS-low group.

Apart from impacting the immune microenvironment and anti-tumor immune response, amino acid metabolism also plays an essential role in driving drug resistance (21). Herein, we detected the sensitivity of the different AAMRGS subgroups to chemotherapy drugs and molecularly targeted drugs. The CGP database was used to predict the IC50 value of each drug for the two subgroups. We found that samples in the AAMRGS-high group were more sensitive to bleomycin, bortezomib, doxorubicin, gemcitabine, and paclitaxel (**Figure 8A**). Meanwhile, samples with low AAMRGS scores showed higher sensitivity to axitinib, bosutinib, cyclopamine, dasatinib, docetaxel, erlotinib, gefitinib, nilotinib, and rapamycin (**Figure 8B**). These results indicated that there existed substantial heterogeneity between the two subgroups, and the AAMRGS score might be a great indicator for predicting drug responses.

### The expression of three AAMRGs in cells and cell proliferation under drug treatment

**Figure 9:**
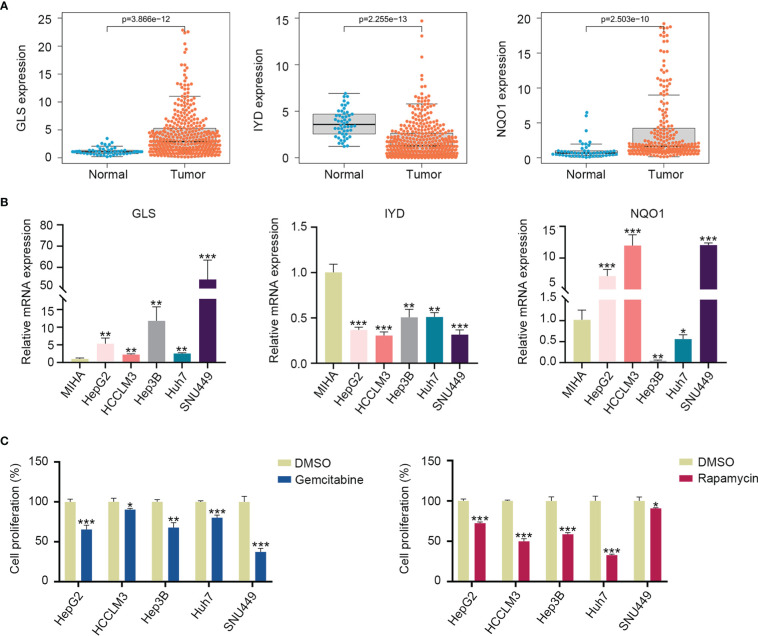
Three signature gene expressions and cell proliferation under drug treatment. **(A)** Expression of GLS, IYD, and NQO1 between the HCC tissues and normal tissues in the TCGA database. **(B)** Validation of the GLS, IYD, and NQO1 expression in the normal liver cell and liver cancer cells by quantitative real-time PCR. **(C)** The cell proliferation rate of liver cancer cells after 48h of rapamycin or gemcitabine treatment. *P < 0.05, **P < 0.01, ***P < 0.001.

According to the TCGA and ICGC cohorts, the expressions of GLS and NQO1 were upregulated, and IYD expression was downregulated in the HCC tissues compared with normal tissues (**Figure 9A**, **Supplementary Figure S5**). To validate the expression of three signature genes, we examined the mRNA expression of these genes in the normal liver cell MIHA and liver cancer cells. The results showed that GLS expression was elevated in HepG2, HCCLM3, Hep3B, Huh7, and SNU449 cell lines compared with MIHA. The expression of NQO1 was increased in HepG2, HCCLM3, and SNU449, while it decreased in Hep3B and Huh7. IYD expression by HepG2, HCCLM3, Hep3B, Huh7, and SNU449 cell lines was significantly lower than that in MIHA (**Figure 9B**). It could be seen that the mRNA expression level of the three signature genes varied among the five liver cancer cell lines. Then, we measured the proliferation capability of these liver cancer cells under the predicted drug treatment. The CCK8 assay showed that gemcitabine significantly reduced SNU449 cell proliferation, and rapamycin remarkedly inhibited the proliferative ability of Huh7 (**Figure 9C**).

## Discussion

Metabolic reprogramming is one of the most features of cancer (22). Tumor cells alter metabolic patterns to meet their increased nutritional requirements for exponential growth and proliferation (23). Although it is widely accepted that dysregulated glucose metabolism is prevailing in many cancer types, the increased demand for amino acids is also necessary to sustain cell proliferation and tumor progression. Amino acids are used not only to synthesize proteins but also to produce energy or to convert them into other physiologically active substances (24). Aberrant amino acid metabolism has been reported to play a crucial role in malignant biological behaviors of tumors and treatment resistance (25, 26). As the major metabolic organ, the liver is essential in regulating metabolism homeostasis. Alterations in hepatic metabolism drive the development and progression of HCC. Previous studies have identified glucose metabolism-related signatures for HCC to predict prognosis and tumor microenvironment (27, 28). However, there are few studies focused on amino acid metabolism. Systematically elucidating the metabolic characteristics of amino acids in HCC is vital for understanding the mechanisms of pathogenesis to improve cancer therapy.

In this study, we concentrated on amino acid metabolism and identified an AAMRGS to predict the prognosis, immune microenvironment, and therapeutic sensitivity of HCC. Differing from traditional approaches, we applied the NMF consensus clustering analysis to identify amino acid metabolism-related molecular subtypes based on 374 hallmark genes of amino acid metabolism. Notably, NMF has become one of the most potent clusterings and feature selection methods. NMF provides an efficient dimension reduction approach that assists in precisely identifying molecular subtypes (29). According to the results of NMF analysis, the HCC samples were clustered into two subgroups, including C1 and C2, which showed significant differences in prognosis and molecular characteristics. In GSVA analysis, signaling pathways related to amino acid metabolism and hallmark gene sets associated with metabolism were markedly enriched in C2. The above results verified that there was heterogeneity with regard to amino acid metabolism between the two subgroups. Then, we compared the differential expression of AAMRGs between the C1 and C2 groups. Based on the differentially expressed AAMRGs, the AAMRGS was constructed through Cox regression analysis and the LASSO algorithm. Three essential genes were included in this signature (GLS, IYD, and NQO1). The expressions of GLS and NQO1 were significantly higher in TCGA and ICGC cancer tissues, while IYD expression was significantly lower in HCC tissues compared with normal tissues. We also verified the expression of these genes in the HCC cell line. GLS encodes a phosphate-activated amidohydrolase, a vital enzyme involved in the modulation of glutamine metabolism. It has been reported that aberrant GLS expression promotes cancer cell proliferation (30). IYD is the crucial regulator of the iodotyrosine metabolism pathway and is associated with thyroid disease (31). NQO1, a member of the NAD(P)H dehydrogenase family, has an important function in redox processes and shows high expression in a variety of solid tumors (32). Compared with the single gene, our risk signature consisted of these three AAMRGs (GLS, IYD, and NQO1) showed accurate predicting ability. Remarkably, the AAMRGS could distinguish between high- and low-risk populations and was an independent risk factor for prognosis. Besides, the AAMRGS score had close correlations with clinical features (pathological grade, T staging, clinical-stage, vascular invasion, and virus infection), indicating that the AAMRGS was involved in tumorigenesis and progression, making it a predictive biomarker with high clinical value.

Except for the prognostic value of the AAMRGS, we further explore the intrinsic molecular characteristics of the AAMRGS subgroups. The missense mutation was most common in HCC, as previously reported (33). The genome mutation profile showed that there were great differences in gene mutation probabilities between the two AAMRGS subgroups. In the AAMRGS-high group, the rate of mutation in TP53 was greatest, up to 35%, compared with only 21% in the AAMRGS-low group. It is clear that TP53 is the hotspot mutation in all cancers. TP53 mutation contributes to carcinogenesis and tumor development (34). Currently, many studies revealed the critical roles of TP53 in regulating cellular amino acid metabolism. P53 protein, encoded by TP53, protects the cell from metabolic stress and facilitates tumor cell survival by promoting aspartate and serine synthesis to produce energy (35, 36). Besides, CTNNB1 had a higher probability of mutation in the AAMRGS-low group. The mutation of CTNNB1 has been implicated in controlling tumor cell proliferation, differentiation, and progression, which due to its mutation, led to abnormal activation of the Wnt/β-catenin signaling pathway (37). Nevertheless, there were no studies about the function of CTNNB1 in metabolism. Consequently, the poorer prognosis in the AAMRGS-high group than in the AAMRGS-low group might owe to the high TP53 mutation.

Next, we compared the differences in immune cell infiltration between the two subgroups. The results of our study indicated that the proportions of Tfh cells, Tregs, M0 macrophages, M2 macrophages, and neutrophils infiltration were significantly increased in the AAMRGS-high group than in the AAMRGS-low group. It is well known that Tregs and M2 macrophages have negative regulation on anti-tumor immunity. As the predominant suppressor cells of the immune system, Tregs promote the M2-like tumor-associated macrophages accumulation in the TME by inhibiting IFNγ from CD8^+^ T cells, which enhances their metabolic fitness and pro-tumor gene expression (38). Besides, the high infiltration of Tregs contributes to tumor malignancy and is associated with poor prognoses (39, 40). The regulator effects of macrophages are a double-edged sword, depending on their polarization state. Proinflammatory M1 macrophages have anti-tumor properties, while anti-inflammatory M2 macrophages inhibit anti-tumor immunity and promote tumor growth (41). Previous studies reported the vital role of Hedgehog signaling in regulating the metabolism and energy consumption of M2 macrophages (42). Moreover, macrophages polarization towards the M1 state is enhanced, and that towards the M2 state is reduced under amino acid deficiency conditions. Except for the macrophages, Tfh cells and neutrophils have also been reported to be affected by microenvironmental metabolism (43, 44). Notably, the infiltration of effector cells of anti-tumor immune response was higher in the AAMRGS-low group, such as CD8^+^ T cells, activated memory CD4^+^ T cells, activated NK cells, monocytes, and mast cells. Consistently, the results of ssGSEA analysis also indicated the two subgroups differed significantly in immune cells, immune function, and related signaling pathways. From these results, it could be seen that amino acid metabolism had a vital influence on anti-tumor immunity. Therefore, our AAMRGS score could provide worthy information to predict immune cell infiltration and functional status in the TIME.

Individualized treatment targeting molecular and immune characteristics is beneficial in improving the clinical outcomes of HCC (45). Notably, immune checkpoint inhibitors have shown great promise for curing malignancies. Antibody agents, such as PD1/PD-L1 and CTLA4 inhibitors, demonstrate robust and durable clinical responses (46). However, the therapeutic efficiency of immunotherapy is not yet satisfactory, especially in tumors with a low mutation burden. The major unresolved challenge in immunotherapy for HCC is discovering and validating predictive biomarkers (4). In our study, the AAMRGS score exhibited positive correlations with immune checkpoints. The patients with high AAMRGS scores had higher expression of PD1, CTLA4, TIM3, CD96, and TIGIT, indicating that patients in the AAMRGS-high group might be more sensitive to immune checkpoint inhibitors. Meanwhile, the two AAMRGS subgroups showed different drug sensitivity to chemotherapeutic and molecularly targeted drugs. It is worth noting that amino acid metabolism plays a vital role in anti-tumor immune response and drug resistance (47). For example, the combination therapy of arginine metabolizing enzymes and immune checkpoint inhibitors increases intratumoral MHC expression and increases the presence of M1 phenotype macrophages, resulting in synergistic anti-tumor effects (48). Wang et al. reported that the metabolic reprogramming of amino acids is involved in the resistance of tumor cells to tyrosine kinase inhibitors (49). In brief, the AAMRGS had a high value in guiding individualized therapy. However, our study was based on public databases. We simply verified the differential mRNA expression of the three signature genes between the normal liver cell and HCC cells. Clinically, gemcitabine is a commonly used drug for cancer treatment (50, 51). Moreover, many kinds of literature have reported that rapamycin displays anti-tumor activity in patients (52, 53). So, we examined the inhibitory effect of these two predicted chemotherapy drugs on different liver cancer cells. The different HCC cell lines displayed different drug sensitivity to predicted drugs. We speculated that this discrepancy could be due to different expression levels of the three key genes. Due to the highly heterogeneous characteristics of HCC, the intra-tumor and surrounding microenvironment vary significantly from one sample to another sample. As a result, our signature needs to be validated in a large-scale, multicenter, prospective study.

In conclusion, we identified a novel AAMRGS and comprehensively analyzed its role in prognosis, immune microenvironment, and treatment sensitivity. The AAMRGS could discriminate the molecular and clinical features of HCC. Besides, the AAMRGS was an independent risk factor for prognosis. More importantly, there was a close relationship between the AAMRGS and the immune microenvironment. The immune cell infiltration and immune checkpoint expression could be predicted by the AAMRGS, which might reflect anti-tumor immunity and sensitivity to individualized therapy. Thus, our AAMRGS was a robust and promising biomarker in predicting clinical outcomes, immune status, and therapeutic sensitivity of HCC.

## Data availability statement

Publicly available datasets were analyzed in this study. This data can be found here: TCGA: https://portal.gdc.cancer.gov/; ICGC: https://dcc.icgc.org/releases/current/Projects/LIRI-JP.

## Author contributions

KT and XL conceived and designed the experiments. YL, HM, SJ, JW and YM analyzed the data. YL and KT wrote the paper. All authors read and approved the final manuscript.
