# Supplementary material for: Comprehensive analysis of the amino acid metabolism-related gene signature for prognosis, tumor immune microenvironment, and candidate drugs in hepatocellular carcinoma
Source: Front Immunol. 2022 Dec 13;13:1066773. doi: 10.3389/fimmu.2022.1066773 (PMC9792509; doi:10.3389/fimmu.2022.1066773)
Supplement: Supplementary file 1 [file DataSheet_1.docx]

**
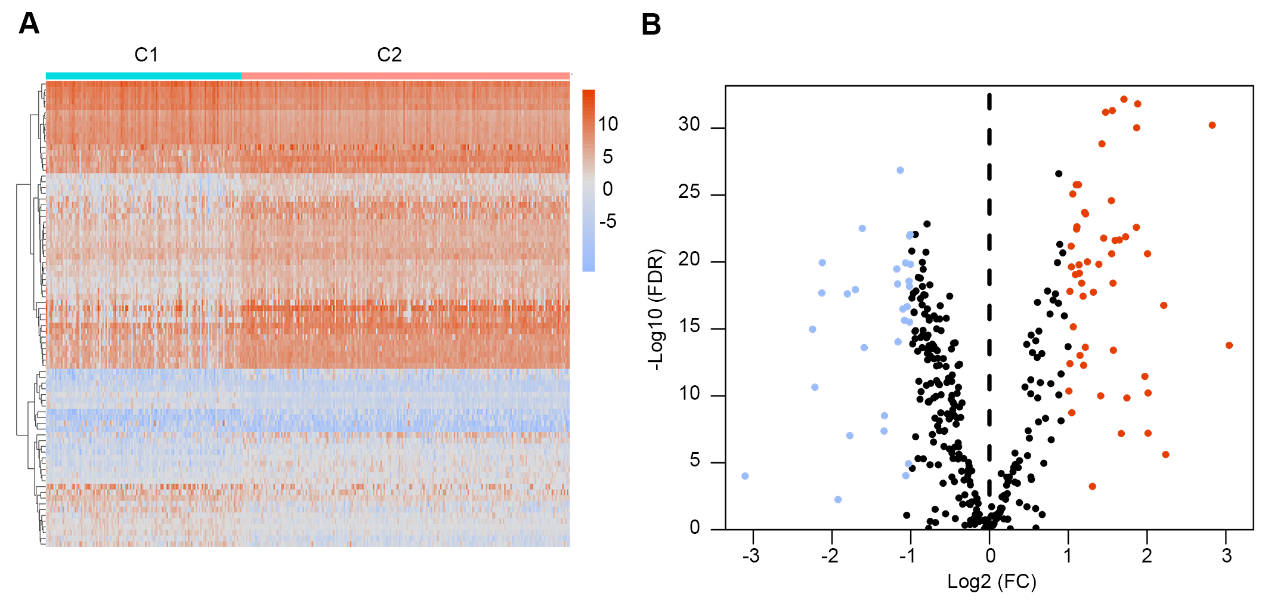
**

**Supplementary Figure S1.** Differentially expressed AAMRGs between the molecular subtypes. (A) Heatmap. (B) Volcano plot.


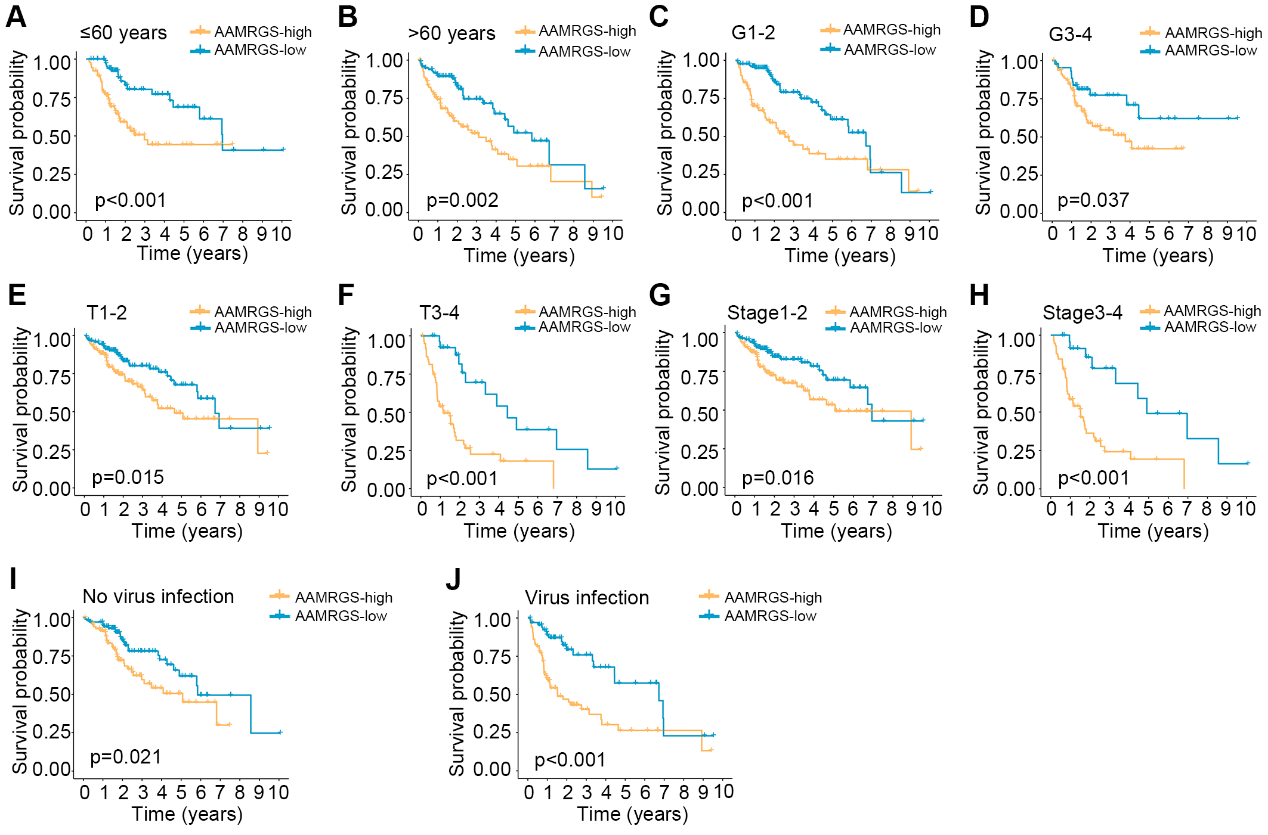


**Supplementary Figure S2.** KM survival analysis of the subgroup of clinicopathological factors. (A) ≤ 60 years. (B) > 60 years. (C) Grade 1–2. (D) Grade 3–4. (E) T stage 1–2. (F) T stage 3–4. (G) Clinical staging 1-2. (H) Clinical staging 3-4. (I) No virus infection. (J) Virus infection.

**
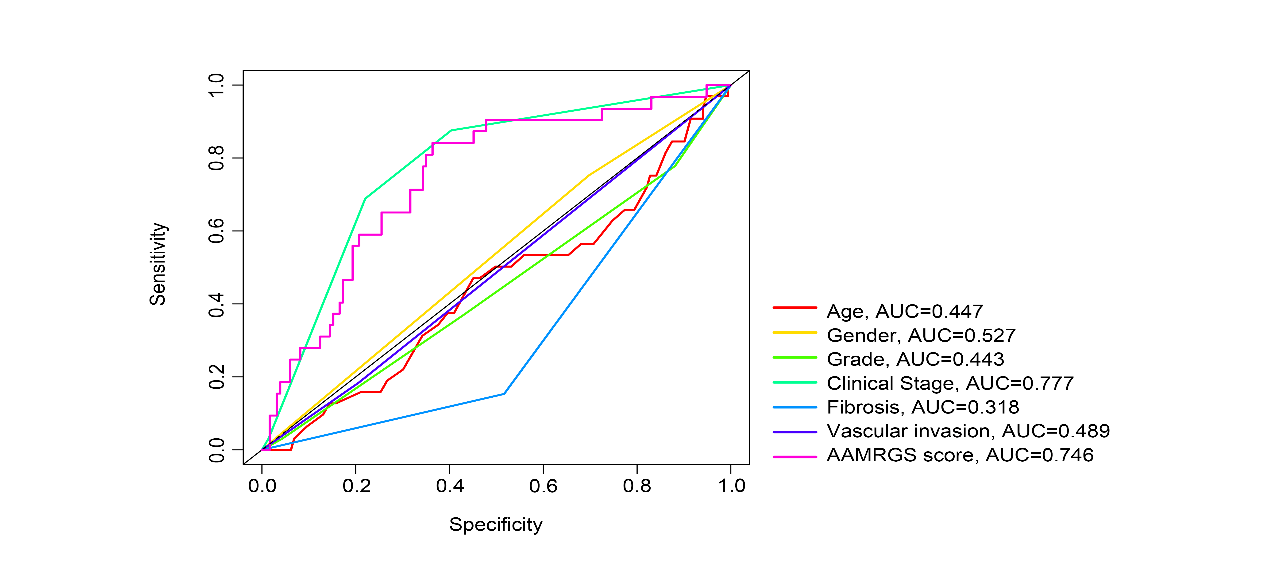
**

**Supplementary Figure S3.** The ROC curve analysis of AAMRGS score and other clinical factors for predicting 1-year OS.

**
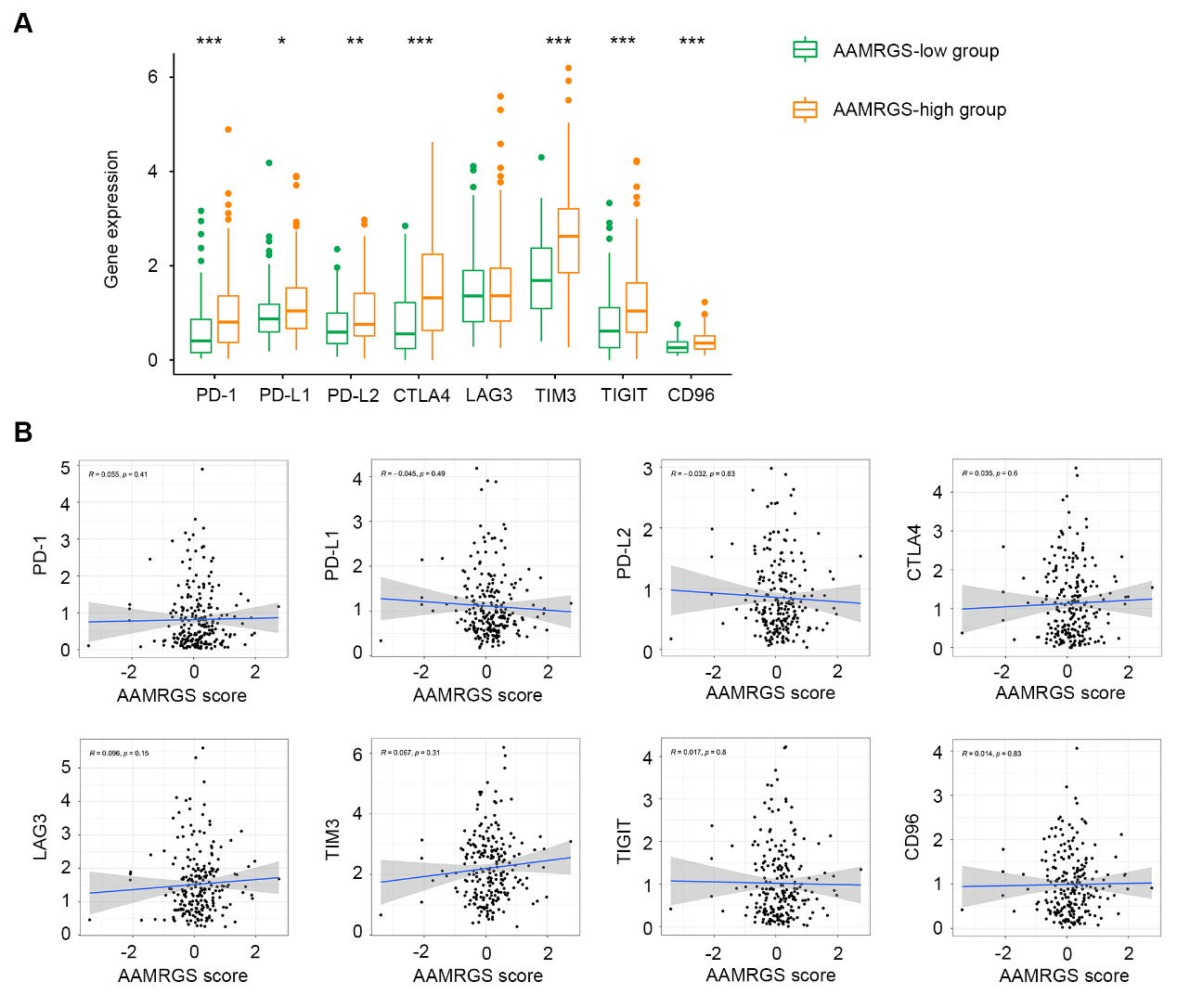
**

**Supplementary Figure S4.** Association of the AAMRGS scores with immune checkpoints in the ICGC database. (A) Differential expression of immune checkpoints between the AAMRGS-high and AAMRGS-low groups. (B) Correlation analysis of immune checkpoints and the AAMRGS scores. * P < 0.05, ** P < 0.01, *** P < 0.001.


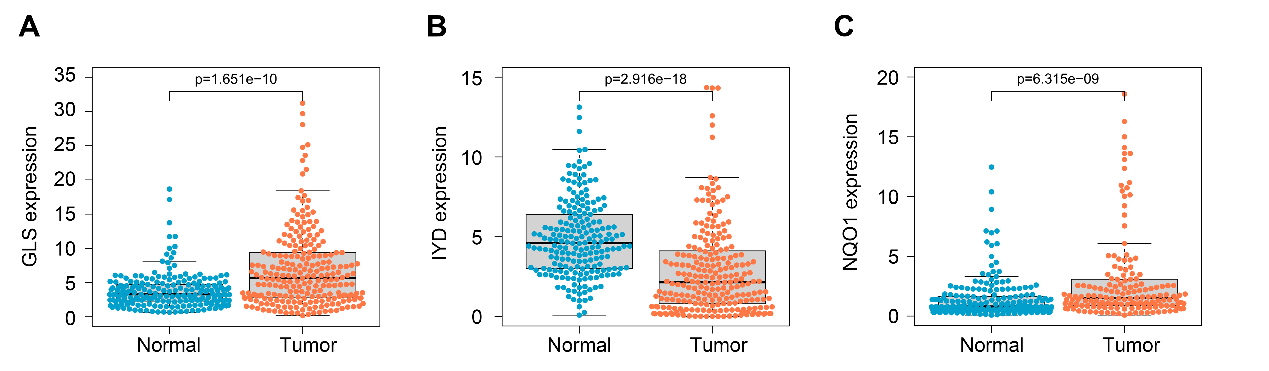


**Supplementary Figure S5.** Expression of GLS, IYD, and NQO1 between the HCC tissues and normal tissues in the ICGC database. (A) GLS expression. (B) IYD expression. (C) NQO1 expression.

**Supplementary Table S1** Primer sequences of quantitative real-time PCR

| **Gene** | **Forward primer** | **Reverse primer** |
| --- | --- | --- |
| GLS | CTGGAGGAAAGGTTGCAGATTA | GAATGCCTCTGTCCATCTACTG |
| IYD | CCCTTCTCTCATAACCACTATCC | CCTGACTGACCGTCTCTTATTG |
| NQO1 | GGACCTCTATGCCATGAACTTCA | CAGAACAGACTCGGCAGGATAC |
| GAPDH | ACATCGCTCAGACACCATG | TGTAGTTGAGGTCAATGAAGGG |

**Supplementary Table S2** Univariate Cox regression analysis of AAMRGs

| **Gene** | **HR** | **HR.95L** | **HR.95H** | **P-value** |
| --- | --- | --- | --- | --- |
| TXNRD1 | 1.0148 | 1.0102 | 1.0194 | 0.0000 |
| PSMD14 | 1.1109 | 1.0741 | 1.1488 | 0.0000 |
| PSMD1 | 1.0842 | 1.0562 | 1.1130 | 0.0000 |
| SMS | 1.0273 | 1.0174 | 1.0374 | 0.0000 |
| AMD1 | 1.1573 | 1.0973 | 1.2205 | 0.0000 |
| PSMA1 | 1.0739 | 1.0453 | 1.1033 | 0.0000 |
| IARS1 | 1.0848 | 1.0508 | 1.1199 | 0.0000 |
| RARS1 | 1.0791 | 1.0469 | 1.1123 | 0.0000 |
| GSR | 1.0200 | 1.0119 | 1.0281 | 0.0000 |
| PSMB2 | 1.0355 | 1.0210 | 1.0502 | 0.0000 |
| MARS1 | 1.0780 | 1.0458 | 1.1112 | 0.0000 |
| DLAT | 1.0728 | 1.0417 | 1.1049 | 0.0000 |
| SLC44A1 | 1.1406 | 1.0774 | 1.2076 | 0.0000 |
| LARS1 | 1.1049 | 1.0579 | 1.1540 | 0.0000 |
| PSMD6 | 1.2409 | 1.1285 | 1.3645 | 0.0000 |
| PSMD2 | 1.0230 | 1.0128 | 1.0332 | 0.0000 |
| PSME3 | 1.0513 | 1.0270 | 1.0762 | 0.0000 |
| GOT2 | 0.9872 | 0.9810 | 0.9933 | 0.0001 |
| DARS1 | 1.0572 | 1.0284 | 1.0868 | 0.0001 |
| SRM | 1.0115 | 1.0057 | 1.0174 | 0.0001 |
| RIMKLA | 2.5737 | 1.5965 | 4.1490 | 0.0001 |
| CSAD | 0.9112 | 0.8693 | 0.9551 | 0.0001 |
| PYCR1 | 1.0216 | 1.0105 | 1.0328 | 0.0001 |
| PSMD11 | 1.0625 | 1.0300 | 1.0959 | 0.0001 |
| GLS | 1.0728 | 1.0347 | 1.1123 | 0.0001 |
| PSMA5 | 1.0305 | 1.0144 | 1.0469 | 0.0002 |
| LIPT1 | 1.4572 | 1.1947 | 1.7775 | 0.0002 |
| PSMD7 | 1.0500 | 1.0233 | 1.0774 | 0.0002 |
| SMOX | 1.0490 | 1.0228 | 1.0759 | 0.0002 |
| PSMA3 | 1.0384 | 1.0177 | 1.0596 | 0.0003 |
| ENOPH1 | 1.0593 | 1.0267 | 1.0929 | 0.0003 |
| PSME4 | 1.1342 | 1.0566 | 1.2175 | 0.0005 |
| PAPSS1 | 1.0695 | 1.0298 | 1.1107 | 0.0005 |
| PSMD13 | 1.0285 | 1.0123 | 1.0450 | 0.0005 |
| ADO | 1.1894 | 1.0782 | 1.3121 | 0.0005 |
| IVD | 0.9670 | 0.9485 | 0.9857 | 0.0006 |
| HAO1 | 0.9947 | 0.9916 | 0.9977 | 0.0007 |
| ACAT1 | 0.9868 | 0.9792 | 0.9944 | 0.0007 |
| PSMD10 | 1.0441 | 1.0183 | 1.0705 | 0.0007 |
| NQO1 | 1.0020 | 1.0008 | 1.0032 | 0.0009 |
| PSPH | 1.0297 | 1.0121 | 1.0477 | 0.0009 |
| SARS1 | 1.0152 | 1.0062 | 1.0244 | 0.0010 |
| KARS1 | 1.0271 | 1.0108 | 1.0437 | 0.0011 |
| PSMD9 | 1.3066 | 1.1132 | 1.5336 | 0.0011 |
| PSMD12 | 1.0715 | 1.0280 | 1.1169 | 0.0011 |
| IYD | 0.8280 | 0.7370 | 0.9302 | 0.0015 |
| EEF1E1 | 1.0881 | 1.0329 | 1.1463 | 0.0015 |
| DMGDH | 0.9748 | 0.9594 | 0.9904 | 0.0016 |
| PSMB5 | 1.0127 | 1.0045 | 1.0210 | 0.0023 |
| CDO1 | 0.9942 | 0.9904 | 0.9979 | 0.0024 |
| EPRS1 | 1.0247 | 1.0086 | 1.0411 | 0.0025 |
| PAH | 0.9951 | 0.9918 | 0.9983 | 0.0028 |
| RPL17 | 1.0281 | 1.0094 | 1.0471 | 0.0031 |
| MTAP | 1.4755 | 1.1381 | 1.9129 | 0.0033 |
| AZIN2 | 2.5413 | 1.3603 | 4.7476 | 0.0034 |
| PSMA7 | 1.0051 | 1.0017 | 1.0086 | 0.0036 |
| RPS8 | 1.0014 | 1.0004 | 1.0023 | 0.0038 |
| PDHA2 | 1869.5266 | 11.4204 | 306043.8382 | 0.0038 |
| SCLY | 1.9259 | 1.2344 | 3.0048 | 0.0039 |
| PHGDH | 1.0144 | 1.0045 | 1.0244 | 0.0044 |
| PSMC6 | 1.1260 | 1.0375 | 1.2220 | 0.0045 |
| ASNS | 1.0572 | 1.0167 | 1.0993 | 0.0052 |
| SERINC2 | 1.0039 | 1.0011 | 1.0067 | 0.0055 |
| GRHPR | 0.9893 | 0.9817 | 0.9969 | 0.0059 |
| OTC | 0.9945 | 0.9906 | 0.9985 | 0.0063 |
| FTCD | 0.9957 | 0.9927 | 0.9988 | 0.0067 |
| ADI1 | 0.9934 | 0.9886 | 0.9982 | 0.0073 |
| NAALAD2 | 1.5579 | 1.1264 | 2.1549 | 0.0074 |
| SUOX | 0.9361 | 0.8918 | 0.9827 | 0.0077 |
| GATM | 0.9974 | 0.9955 | 0.9993 | 0.0081 |
| SLC36A4 | 1.4870 | 1.1077 | 1.9962 | 0.0083 |
| RPL5 | 1.0017 | 1.0004 | 1.0029 | 0.0086 |
| BCAT1 | 1.0702 | 1.0167 | 1.1265 | 0.0095 |
| PSMC5 | 1.0130 | 1.0031 | 1.0229 | 0.0096 |
| MAT1A | 0.9982 | 0.9969 | 0.9996 | 0.0099 |

**Supplementary Table S3** Univariate Cox regression analysis of differentially expressed AAMRGs

| **Gene** | **HR** | **HR.95L** | **HR.95H** | **P-value** |
| --- | --- | --- | --- | --- |
| AGXT2 | 0.9744 | 0.9524 | 0.9969 | 0.0258 |
| ALDH7A1 | 0.9850 | 0.9710 | 0.9993 | 0.0400 |
| ASNS | 1.0572 | 1.0167 | 1.0993 | 0.0052 |
| ASRGL1 | 1.0557 | 1.0124 | 1.1008 | 0.0111 |
| BCAT1 | 1.0702 | 1.0167 | 1.1265 | 0.0095 |
| BHMT2 | 0.9958 | 0.9922 | 0.9995 | 0.0257 |
| CDO1 | 0.9942 | 0.9904 | 0.9979 | 0.0024 |
| CPS1 | 0.9985 | 0.9971 | 0.9999 | 0.0353 |
| DMGDH | 0.9748 | 0.9594 | 0.9904 | 0.0016 |
| EEF1E1 | 1.0881 | 1.0329 | 1.1463 | 0.0015 |
| FTCD | 0.9957 | 0.9927 | 0.9988 | 0.0067 |
| GATM | 0.9974 | 0.9955 | 0.9993 | 0.0081 |
| GCDH | 0.9753 | 0.9549 | 0.9961 | 0.0202 |
| GLS | 1.0728 | 1.0347 | 1.1123 | 0.0001 |
| GPT2 | 0.9915 | 0.9835 | 0.9996 | 0.0403 |
| GRHPR | 0.9893 | 0.9817 | 0.9969 | 0.0059 |
| GSTZ1 | 0.9485 | 0.9013 | 0.9982 | 0.0425 |
| HAO1 | 0.9947 | 0.9916 | 0.9977 | 0.0007 |
| IL4I1 | 1.0344 | 1.0049 | 1.0647 | 0.0218 |
| IYD | 0.8280 | 0.7370 | 0.9302 | 0.0015 |
| MAT1A | 0.9982 | 0.9969 | 0.9996 | 0.0099 |
| NQO1 | 1.0020 | 1.0008 | 1.0032 | 0.0009 |
| OTC | 0.9945 | 0.9906 | 0.9985 | 0.0063 |
| PAH | 0.9951 | 0.9918 | 0.9983 | 0.0028 |
| PIPOX | 0.9955 | 0.9914 | 0.9997 | 0.0352 |
| PYCR1 | 1.0216 | 1.0105 | 1.0328 | 0.0001 |
| SLC25A15 | 0.9870 | 0.9760 | 0.9982 | 0.0228 |
| SMOX | 1.0490 | 1.0228 | 1.0759 | 0.0002 |
